# *Hippocampusjapapigu*, a new species of pygmy seahorse from Japan, with a redescription of *H.pontohi* (Teleostei, Syngnathidae)

**DOI:** 10.3897/zookeys.779.24799

**Published:** 2018-08-02

**Authors:** Graham Short, Richard Smith, Hiroyuki Motomura, David Harasti, Healy Hamilton

**Affiliations:** 1 California Academy of Sciences, San Francisco, United States of America California Academy of Sciences San Francisco United States of America; 2 iSeahorse, IUCN Seahorse, Pipefish Stickleback Specialist Group, London U.K. Pipefish Stickleback Specialist Group London United Kingdom; 3 Kagoshima University Museum, Japan Kagoshima University Museum Kagoshima Japan; 4 Port Stephens Fisheries Institute, NSW, Australia Port Stephens Fisheries Institute Nelson Bay Australia; 5 NatureServe, Arlington, Virginia, United States of America Kagoshima University Museum Kagoshima United States of America

**Keywords:** Acanthomorpha, computed tomography, reef fish, new species, systematics, taxonomy, systematics, computed tomography

## Abstract

The pygmy seahorse *Hippocampusjapapigu***sp. n.** is described based on three specimens, 13.9–16.3 mm SL, collected from a mixed soft coral and algae reef at 11 m depth at Hachijo-jima Island, Izu Islands, Japan. The new taxon shares morphological synapomorphies with the previously described central Indo-Pacific pygmy seahorses, *H.colemani*, *H.pontohi, H.satomiae*, and *H.waleananus*, including extremely small size, 12 trunk rings, strongly raised continuous cleithral ring, snout spine, large spine on the eighth lateral and fifth and 12 superior trunk ridges, respectively, and unusual wing-like-protrusions immediately posterior to the head. *Hippocampusjapapigu***sp. n.** can be distinguished from all congeners by the following combination of features in the anterodorsal area of the trunk: bilaterally paired wing-like protrusions formed by a single pair of large, truncate spines projecting dorsolaterad on the first superior trunk ridge, followed by a unique elevated dorsal ridge formed by triangular bony mounds dorsally on the second to fourth superior trunk ridges. In contrast, *H.pontohi* possesses a pair of large truncate spines projecting strongly laterad on both the first and second superior trunk ridges followed by flat surfaces dorsally on the third and fourth superior trunk rings. The new species can be further differentiated by genetic divergence from *H.pontohi* (an uncorrected p-distance of 10.1% in the mitochondrial COI gene) and a striking reticulated white and brown lattice pattern on the head, trunk, and tail. *Hippocampusjapapigu***sp. n.** represents the fifth species of pygmy seahorse recorded in Japan.

## Introduction

The family Syngnathidae contains 57 valid genera and 300 described predominantly small-bodied and cryptic marine species (Dawson 1985; Froese and Pauly 2018), including the seahorses, pipefishes, pipehorses, and seadragons. The family occurs worldwide in shallow temperate to tropical waters in a range of habitats, including seagrass beds, estuaries, coral and rocky reefs, and mangroves (Foster and Vincent 2004; Kuiter 2009; Froese and Pauly 2018). Pygmy seahorses of the genus *Hippocampus* Rafinesque, 1810 are diminutive in size (13.6–26 mm SL), live in close association with octocorals, colonial hydrozoans, bryozoans, seagrass and algae, and are morphologically distinct from the more numerous and larger species (24–350mm SL) of seahorses in possessing a single rather than paired gill openings and trunk brooding of their young (Whitley 1970; Kuiter 2003; Lourie and Randall 2003; Lourie and Kuiter 2008; Gomon and Kuiter 2009; Lourie et al. 2016).

Fundamental information on the taxonomy, systematics, and distribution of pygmy seahorses is still relatively sparse in comparison to the larger seahorse species. While one species, *H.bargibanti* Whitley, 1970, was described in 1970, all other species have been described since 2000. Most are known from very few specimens and only three species have been analyzed genetically (Hamilton et al. 2017). Six pygmy seahorse species are currently recognized and documented throughout the central Indo-Pacific, ranging from the Coral Triangle, West Pacific, Australia, to central Japan: *H.bargibanti*, *H.denise* Lourie & Randall, 2003, *H.colemani* Kuiter, 2003, *H.pontohi* Lourie & Kuiter, 2008, *H.satomiae* Gomon & Kuiter, 2009, and *H.waleananus* Gomon & Kuiter, 2009 (Whitley 1970; Kuiter 2003; Lourie and Randall 2003; Senou et al. 2006, 2007, 2008; Baine and Harasti 2007; Lourie and Kuiter 2008; Motomura et al. 2010; Allen and Erdmann 2012; Smith et al. 2012). Lourie et al.’s (2016) revision of the genus *Hippocampus* informally placed *H.waleananus* in synonymy with *H.satomiae*. However, we recognize the current taxonomic status of *H.waleananus* as valid based on differences in diagnostic morphological characters, including tail ring counts, coronet profile, and body ornamentation (Tables 1, 3), and host association and diurnal versus nocturnal behavior. Another described pygmy species, *Hippocampussevernsi* (Lourie & Kuiter, 2008), was distinguished from *H.pontohi* primarily by features of coloration in life, however the invalidity of this species was subsequently recognised as coloration is not a reliable morphological diagnostic character in seahorses (Lourie et al. 2004, 2008, 2016). Here, as part of this study, we formally synonymize *H.severnsi* under *H.pontohi* based on mitochondrial COI genetic data. All six pygmy species exhibit similar meristic and morphometric characters, and appear to form two natural groupings (Kuiter 2003; Lourie and Kuiter 2008; Gomon and Kuiter 2009). *Hippocampuscolemani*, *H.pontohi*, *H.satomiae* and *H.waleananu*s are morphologically highly conserved, with subtle meristic and morphological differences among these species. These four taxa are united by synapomorphies, including 12 trunk rings, strongly raised continuous cleithral ring, snout spine, large spine on the eighth lateral and fifth and 12^th^ superior trunk ridges, wing-like-protrusions immediately posterior to the head, and associations with a wide range of habitat types. In contrast, *H.bargibanti* and *H.denise* are distinct in overall morphological appearance, including the absence of a distinct coronet and presence of large bulbous tubercles (in place of small spines exhibited by the other pygmy seahorse species), absence of a raised cleithral ring, and exclusive habitat association with gorgonian corals.

Japan is recognized as a global hotspot of marine biodiversity (Roberts et al. 2002; Allen 2008; Tittensor et al. 2010; Mittermeier et al. 2011), with 53 recorded species of syngnathids (Senou 2007; Han et al. 2017; Wibowo and Motomura 2017; Froese and Pauly 2018), including ten species of seahorses (Lourie et al. 2016; Han et al. 2017) of which four are true pygmy seahorses from the widely dispersed subtropical island groups Ryukyu, Ogasawara, and Izu: *H.bargibanti*, *H.denise*, and *H.pontohi*, and a fourth species that appears to be *H.colemani* (Senou et al. 2006, 2007; Motomura et al. 2010; Allen and Erdmann 2012). Inshore surveys of the marine ichthyofauna conducted at Hachijo-jima Island, Izu Islands (Senou et al. 2002), approximately 287 km south of Tokyo, have recorded what appears to be an undescribed species of true pygmy seahorse inhabiting mixed soft coral and algae reefs at shallow depths of 5–22 m, which was first brought to our attention from marine life books (*Hippocampus* sp. 7, Kuiter 2009: 57) and online photographs (Smith 2017) before specimens were acquired. This free-living species has been previously observed by local scuba divers from southern to central-eastern Japan at Kashiwa-jima Island, Sukumo Bay; Kushimoto, Kii Peninsula; Osezaki, Izu Peninsula; the Izu islands of Miyake and Hachijo; Sagami Bay; and Chichi-jima, Ogasawara Islands. The new taxon is meristically and morphologically most similar to *H.pontohi* (Tables 1, 3); however, closer examination of a type specimen employing micro-computed tomography (μCT) reveals notable internal differences from a non-type specimen of its congener. Distinguishing characters include the anterodorsal area of the trunk, where bilaterally paired wing-like protrusions are formed by a single pair of large truncate spines projecting dorsolaterad, followed by an elevated dorsal ridge formed by unusual triangular bony mounds. The elevated dorsal ridge is unknown from any other species of seahorse and serves as a key diagnostic morphological character distinguishing *H.japapigu* sp. n. from *H.pontohi*. We can therefore confirm the presence of a 7^th^ species of pygmy seahorse that we hereby describe as the new species *Hippocampusjapapigu*, so far only found in subtropical southeast Japan.

## Materials and methods

Three specimens of *H.japapigu* were collected with hand nets while scuba diving in less than 15 m depth. Counts and measurements were performed on high-resolution digital images of specimens using ImageJ (Rasband et al. 1997) to the nearest 0.01 mm following Lourie and Randall (2003) and Lourie and Kuiter (2008). External morphological characters were documented using a dissecting microscope. Live specimens of *H.japapigu* and *H.pontohi* photographed in situ were used to make morphological comparisons of external diagnostic characters only and were not collected as part of this study.

In order to document internal morphological characters, the axial skeleton was examined via non-destructive x-ray micro-computed tomography (μCT) scans at the Karel F. Liem Bioimaging Facility (Friday Harbor Laboratories, University of Washington) using a Bruker Skyscan 1173 scanner (Billerica, MA) with a 1 mm aluminum filter at 60 kV and 110 lA on a 2048 3 2048 pixel CCD at a resolution of 8.8 lm. The specimens were placed inside a 50 ml plastic Falcon tube (Corning, NY), supported by two thin foam pads to prevent movement during scanning and wrapped in ethanol (70%)-infused cheesecloth to prevent desiccation. The resulting CT data were visualized, segmented, and rendered in Horos software (www.horosproject.org).

The holotype (UW 157506) and one paratype (UW 157507) were deposited in the fish collection of the Burke Museum at the University of Washington, the second paratype (KAUM-I. 111770) was deposited at the Kagoshima University Museum (KAUM), and comparative material (one non-type specimen of *Hippocampuspontohi*, AMS I.47833-001, male) was obtained from the Australian Museum (AMS) fish collection. A segment of the mitochondrial cytochrome c oxidase subunit I (COI) DNA was sequenced from the *H.japapigu* paratype (KAUM-I. 111770). DNA extraction, PCR amplification, alignment, and analysis of COI sequence was performed following protocols described in Hamilton et al. (2017). Genetic distances (uncorrected *p*-distances) were calculated based on COI using MEGA v. 7.0.26 (Kumar et al. 2017).

## Taxonomy

### Hippocampus japapigu sp. n.

Taxon classificationAnimaliaSyngnathiformesSyngnathidae

http://zoobank.org/F3DC73D6-E040-458E-9648-680EBAC55D20

Figures 1 , 2 , 3 , 4 , 5 , 7 , 8 , 9 , Video 1 , Tables 1 , 2

#### Holotype.

**Figure 1.**
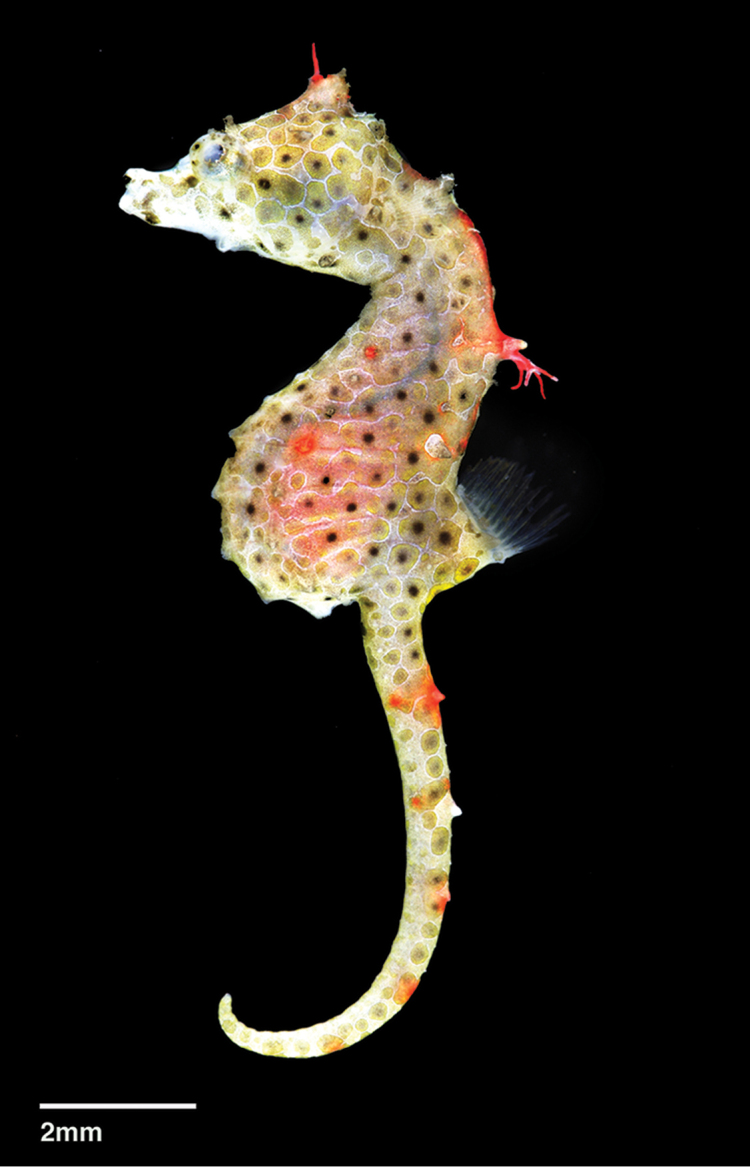
*Hippocampusjapapigu*, UW 157506, female holotype directly after collection, 16.33 mm SL, Hachijo-jima Island, Izu Islands, Japan (photograph Hiroyuki Motomura).

UW 157506, Fig. 1, 16.27 mm SL, off Imasaki, Okago, Hachijo-jima Island, Izu Islands, Japan, 33°08'48"N, 139°44'37"E, depth 10 m, 18 Aug. 2017, collected by Shoichi Kato using a hand net.

#### Paratypes.

**Figure 2.**
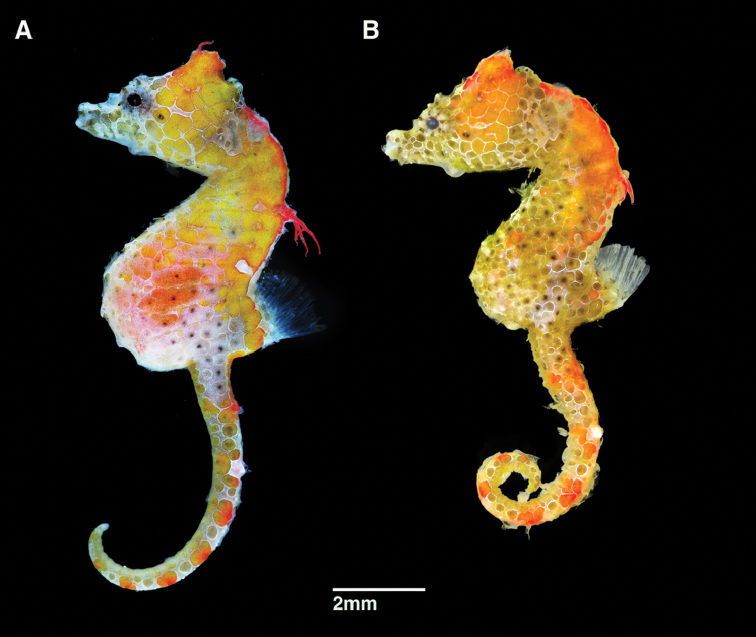
*Hippocampusjapapigu*, paratypes directly after collection (**A**) UW 157507, male, 15.59 mm SL (**B**) KAUM-I. 111770, female, 14.54 mm SL, Hachijo-jima Island, Izu Islands, Japan (photographs Hiroyuki Motomura).

UW 157507, Fig. 2A, 15.59 mm SL, off Imasaki, Okago, Hachijo-jima island, Izu Islands, Japan, 33°08'48"N,139°44'37"E, depth 13 m, 18 Aug. 2017, S. Kato; KAUM – I. 111770, Fig. 2B, 14.54 mm SL, Yaene, Okago, Hachijo-jima Island, Izu Islands, Japan, 33°05'47"N, 139°46'10"E, depth 18 m, 12 Jan. 2018, S. Kato.

#### Other material.

Hachijo-jima Island, Izu Islands, Japan, July 2013, 10 to 20 m depth, two photographs of two individuals, R Smith (Figs 4, 5).

#### Comparative material.

*Hippocampuspontohi* AMS I.47833-001, Fig. 6. Data from *H.bargibanti*, *H.denise*, *H.colemani*, *H.satomiae*, and *H.waleananus* also from Kuiter (2003), Lourie and Kuiter (2008), and Gomon and Kuiter (2009).

**Diagnosis.***Hippocampusjapapigu* sp. n. differs from its congeners by the following combination of characters: tail rings 28; dorsal fin rays 14; pectoral fin rays nine; subdorsal rings four; bilaterally paired wing-like protrusions formed by a pair of large truncate spines projecting laterad on first superior trunk ridge; elevated dorsal ridge formed by unique triangular bony mounds dorsally on second, third, and fourth trunk rings with the posterior mound less pronounced; large and prominent spine projecting laterad on eighth lateral trunk ridge.

#### Description.

**Figure 3.**
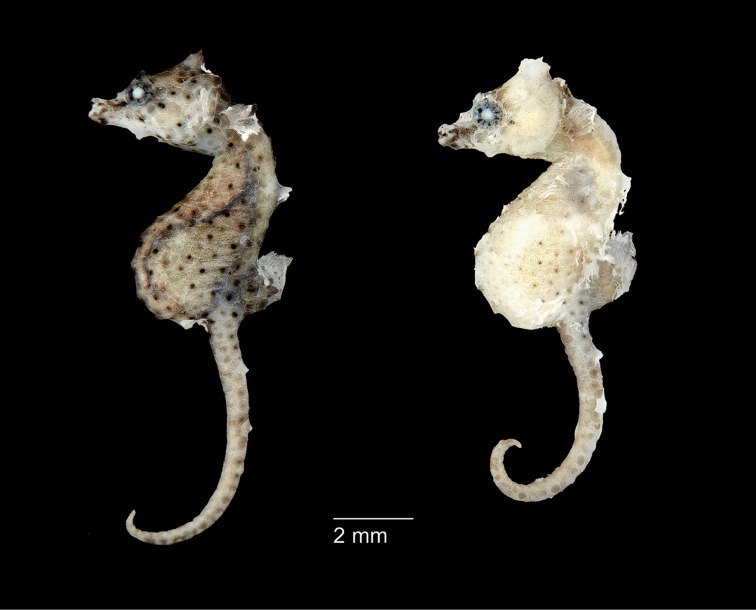
*Hippocampusjapapigu*, UW 157506, preserved female holotype, 16.33 mm SL (left), and UW 157506, 15.59 mm SL, male paratype (right), Hachijo-jima Island, Izu Islands, Japan (photograph Graham Short).

**Figure 4.**
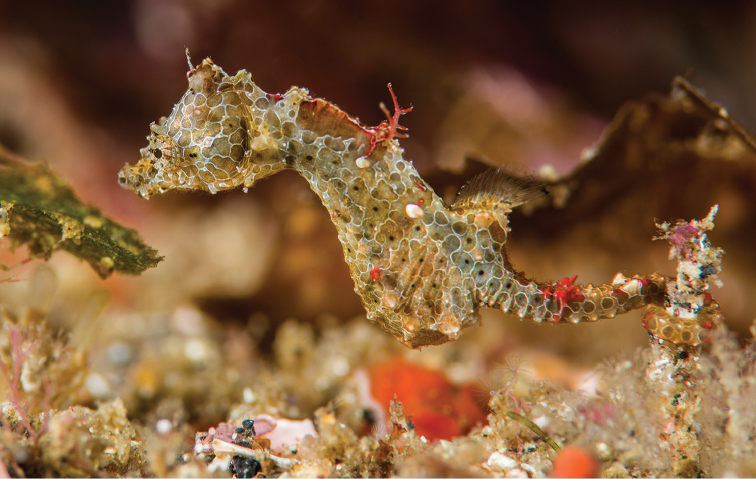
*Hippocampusjapapigu* in situ, Hachijo-jima Island, Izu Islands, Japan at 15 m depth (photograph Richard Smith).

**Figure 5.**
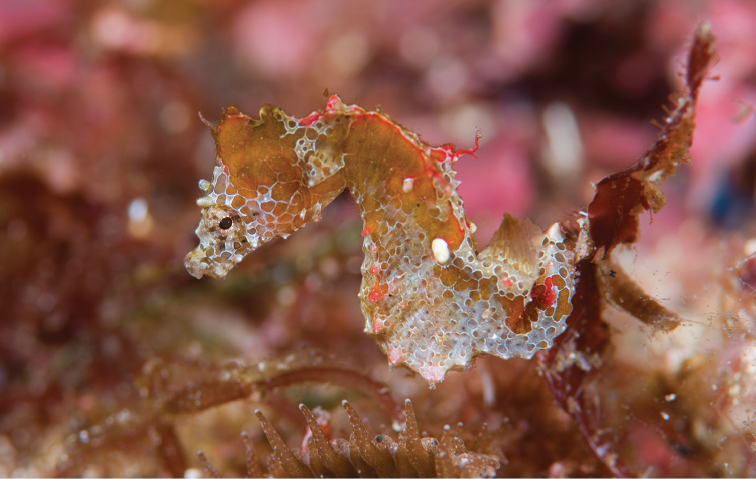
*Hippocampusjapapigu* in situ, Hachijo-jima Island, Izu Islands, Japan from 10 m depth (photograph Richard Smith).

General body shape as in Figs 1–5. Morphometric and meristic characters listed in Table 1. Morphometric data ranges for the three type specimens: Head length 17.9–18.74% in SL, head depth 69.9–72.0% in HL; snout length 27.64–28.7% in HL, bulbous tip absent, snout depth 74.0–85.6% in SnL; post-orbital 55.3–49.9% in HL; distinct, angular coronet, coronet height 58.0–55.9% in HL, unbranched dermal appendage attached to anterior part of coronet; single gill-opening on midline behind coronet supported by elevated cleithral ring; dorsal fin 14 rays; pectoral-fin rays nine; anal fin rays four; trunk rings 12, trunk length 32.49–32.64% in SL, trunk depth just anterior to dorsal fin base 18.4–20.32% in SL; dorsal fin base strongly raised dorsally; subdorsal rings four, dorsal fin base starting immediately posterior to ninth trunk ring and ending immediately posterior to first tail ring; no external pouch visible; tail rings 28, tail length 48.73–49.42 % in SL. Body ornamentation: prominent spine dorsal of eye, small spine ventroposterior to eye; lateral head spine ventral of coronet; two moderately large spines on cleithral ring, upper spine at level of last pectoral fin ray, lower spine at ventral extent of ring; snout spine on midline between eyes; nape spine absent; subdorsal spines four, superior trunk ridge ending with three rounded spines protruding laterally, the posterior spine greatly enlarged on 12^th^ trunk ring; superior trunk ridge with large truncate spines, connected by a solid bony ridge, projecting dorsolaterad on first trunk ring, unique triangular bony mounds arched dorsally on second, third, and fourth trunk rings with the posterior mound less pronounced, trunk appearing denticulate in lateral view, very large bilaterally paired spines on fifth trunk ring, and small spines on sixth trunk ring; lateral trunk ridge with small spine on fifth trunk ring and very large spine on eighth trunk ring; inferior trunk ridge with spines of moderate size beginning on fifth trunk ring and ending on 12^th^ trunk ring; superior tail ridge spines well developed anteriorly, becoming smaller posteriorly, with enlarged spines on fifth, ninth, 12^th^, and 16^th^ tail rings; inferior tail ridge spines absent; caudal fin absent.

**Table 1.** Morphometric measurements and counts of Hippocampus pygmy seahorse species based on holotype specimens. Abbreviations: SnD (snout depth), SnL (snout length), CH (coronet height), HL (head length), HD (head depth), PO (post-orbital length), TrL (trunk length), TaL (tail length), SL (standard length). Numbers separated by a colon represent proportions. Lines present, from top to bottom, numbers for standard length (SL), proportions, and counts for trunk rings, tail rings, dorsal and pectoral fins. The first column is the species holotype.

	* H. japapigu *	* H. pontohi *	* H. colemani *	* H. satomiae *	* H. waleananus *	* H. bargibanti *	* H. denise *
Voucher number/data source	UW 157506	Lourie and Kuiter 2008	Kuiter 2003	Lourie and Kuiter 2008	Gomon and Kuiter 2009	Lourie and Kuiter 2008	Lourie and Kuiter 2008
SL (mm)	16.3	16.7	26.9	13.6	17.8	24.5	15.7
SnD:SnL	74.06	84.2	70.5	86	95.2	100.3	73.6
CH:HL	58.1	47.4	45.6	40.2	48.3	57.3	42.6
HD:HL	69.9	60.6	62.6	51.8	67.9	65.7	48.1
SnL:HL	28.7	23.2	27.7	27	26.8	21.8	32.8
PO:HL	55.3	51.2	52.1	45	51.5	56.9	42.0
HL:SL	18.0	21.7	18.1	22	17.7	16.7	19.9
TrL:SL	32.6	33.3	32.0	30	31.3	27.6	27.7
TaL:SL	49.4	45	50	48	63.4	55.7	52.5
TD9:SL	18.9	13.5	19.2	13	15	12.8	9.3
Trunk rings	12	12	12	12	12	12	12
Tail rings	28	28–30	26–28	27–28	32	31–33	27–28
Dorsal fin rays	14	12	14	13	12	14	13–14
Pectoral Fin rays	9	10	9	9	9	10–11	10–11

**Video 1.** YouTube video of a pair of specimens of *Hippocampusjapapigu* on rocky reef wall (video by Akira Bingoeral 2007)

#### Color in life.

*Hippocampusjapapigu* (Figs 4, 5, and 7) exhibits cryptic coloration: head, trunk and tail, brown, with overlay of reticulate (net-like) irregular quadrilateral and pentagonal skin formations, brown or white, white outline, entire surface of head and body peppered with tiny black dots; elevated dorsal ridge on second to fourth superior trunk rings, engorged red, reticulate color pattern diffuse or absent; dorsal fin base, red, reticulate pattern absent; tail rings with one row of rounded quadrilaterals present, one quadrilateral per ring, brown with white outline; fifth superior ridge spine red; fifth and eighth lateral trunk ridge spines, white; eighth inferior trunk ridge spine, red; fifth, ninth, 12^th^ superior tail ridge spines, red, every fourth ring thereafter with two dorsolateral color spots, red; dermal appendages on coronet anteriorly.

#### Color in alcohol.

Light brown in holotype, pale brown in paratype, with black dots scattered over head, trunk, and anterior to tail.

### Hippocampus pontohi

Taxon classificationAnimaliaSyngnathiformesSyngnathidae

Lourie & Kuiter, 2008

http://zoobank.org/853548F1-CEF4-47CD-8A15-7F225B73BCFC

**Figure 6.**
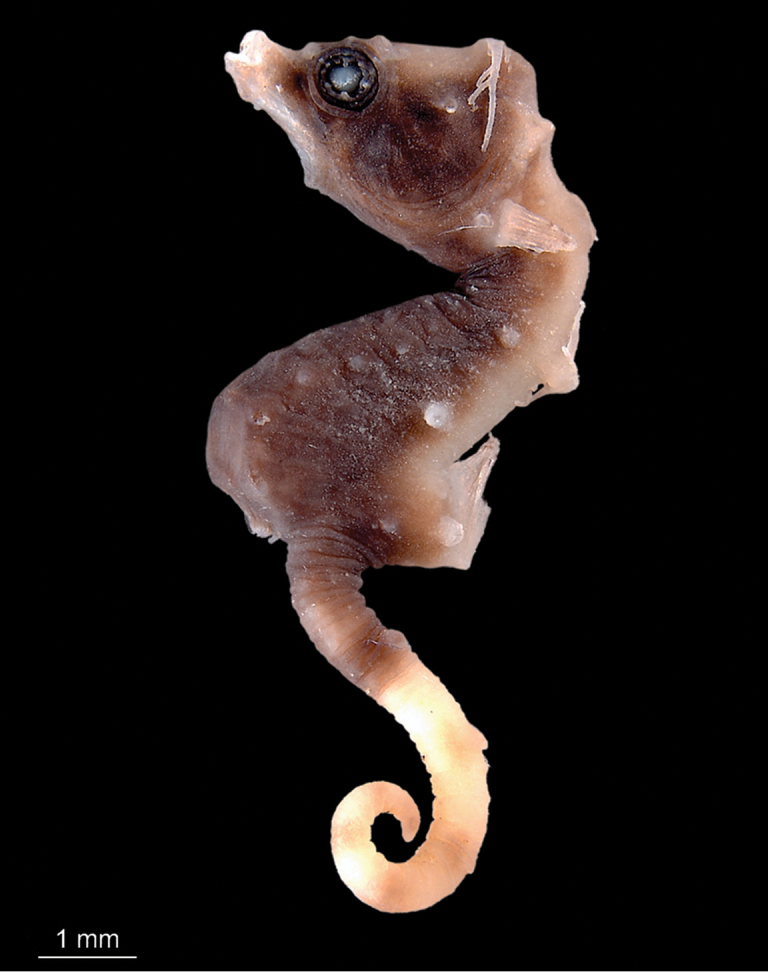
*Hippocampuspontohi*, AMS I.47833-001, preserved male non-type, 13.9 mm SL, Cape Kri, Raja Ampat, Indonesia (photograph Graham Short).

**Figure 7.**
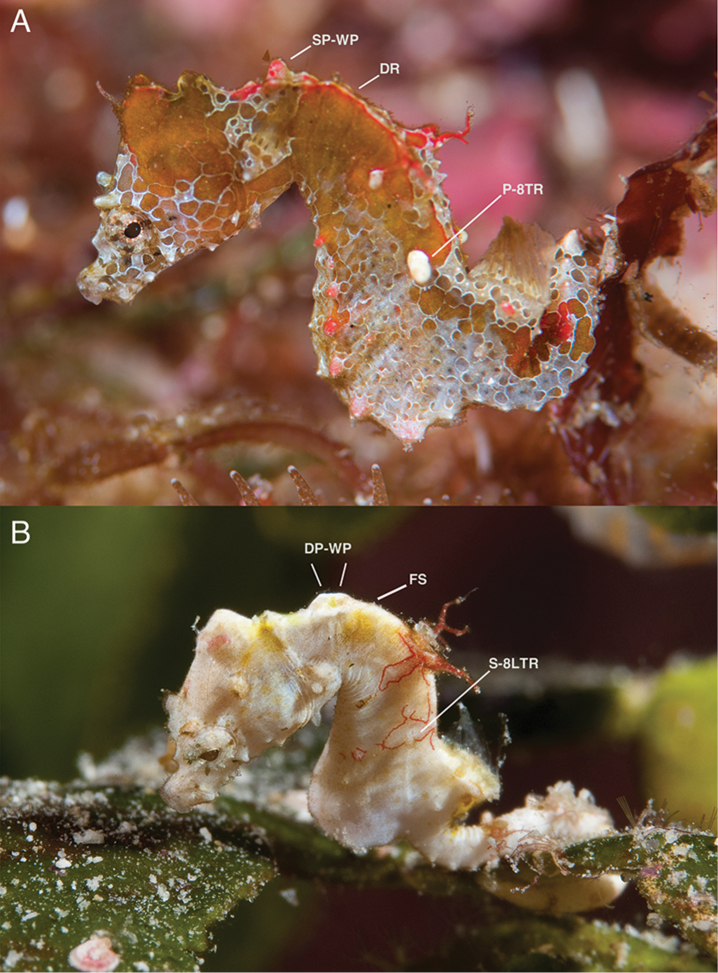
Comparison of live specimens of **A***Hippocampusjapapigu* photographed off Hachijo-jima Island, Japan (Richard Smith), and its most similar congener **B***Hippocampuspontohi* photographed off Tomia Island, southeast Sulawesi, Indonesia (Richard Smith). Note the differences in the anterodorsal area of the trunk in *H.japapigu* vs. *H.pontohi*: single vs. double pair of bilaterally paired wing-like protrusions behind the head, raised dorsal ridge vs. laterodorsal flat surface, and large and prominent vs. small eighth lateral trunk ridge spine. Abbreviations: SP-WP, single pair of bilaterally paired wing-like protrusions; DP-WP, double pair of bilaterally paired wing-like protrusions; DR, raised dorsal ridge; FS, flat dorsal surface; P-8LTR, prominent eighth lateral trunk ridge spine; S-8LTR, small eighth lateral trunk ridge spine.

**Figure 8.**
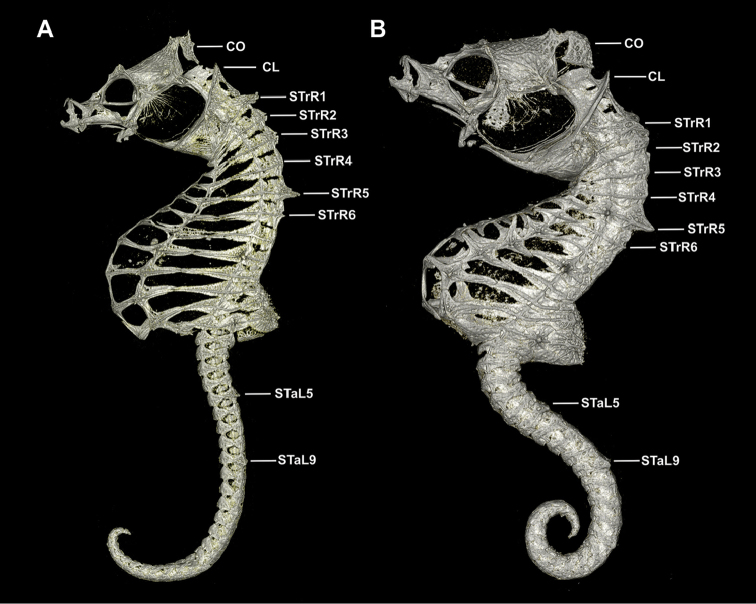
Comparison of the lateral view of reconstructed μCT scans of skeletons of A *Hippocampusjapapigu*, UW 157506, preserved male holotype, 16.33 mm SL, Hachijo-jima Island, Japan, and its most similar congener B *Hippocampuspontohi*, AMS I.47833-001, preserved male non-type 13.9 mm SL, Cape Kri, Raja Ampat, Indonesia (photographs Graham Short).

Figures 6 , 7 , 8 , 10 , Table 1 , 2 Pontoh’s Pygmy Seahorse

Hippocampus severnsi Lourie & Kuiter, 2008: figs. 2B-4B (Bunaken, North Sulawesi, Indonesia); Reijnan et al. 2011: fig. 2B (Siladen I, SE Siladen).

#### Material.

AMS I.47833-001. 13.9 mm SL, GenBank accession number KY066111, Cape Kri, Raja Ampat, Indonesia. 0°33’23.5”S 130°41’25.0”E, depth 6 m, collected by Otto Awom, Gerry Allen, and Mark Erdmann using hand net in small clump of algae and hydroids on vertical surface, 1 January 2007. Mitochondrial COI sequence data and corresponding Genbank accession numbers for additional vouchered specimens of *H.pontohi* (Table 2).

**Table 2.** List of pygmy seahorse specimens, including species, collection locality, voucher number, and COI GenBank accession numbers.

	Species	Locality	Voucher	COI Genbank no.
1	* Hippocamus pontohi *	Indonesia	AM I.47833-001	MH645117
2	* Hippocamus pontohi *	Indonesia	AM I.47831-001	MH645118
3	* Hippocamus pontohi *	Indonesia	AM I.47831-001	MH645119
4	* Hippocamus pontohi *	Indonesia	AM I.47831-003	MH645120
5	* Hippocamus pontohi *	Indonesia	AM I.47831-004	MH645121
6	* Hippocamus pontohi *	Indonesia	AM I.47960-001	MH645122
7	* Hippocamus pontohi *	Indonesia	AM I.47960-002	MH645123
8	* Hippocamus pontohi *	Indonesia	AM I.47832-001	MH645124
9	* Hippocamus pontohi *	Indonesia	AM I.47834-001	MH645125
10	* Hippocamus pontohi *	Indonesia	AM I.47834-002	MH645126
11	* Hippocamus pontohi *	Indonesia	AM I.47834-003	MH645127
12	* Hippocamus pontohi *	Indonesia	AM I.47834-004	MH645128
13	* Hippocamus pontohi *	Indonesia	MZB 3597	KY066111
14	* Hippocampus severnsi *	Indonesia	AM I.47960-003	MH645129
15	* Hippocampus severnsi *	Indonesia	AM I.47960-004	MH645130
16	* Hippocampus severnsi *	Indonesia	AM I.47960-005	MH645131
17	* Hippocampus severnsi *	Indonesia	AM I.47961-001	MH645132
18	* Hippocampus severnsi *	Indonesia	AM I.47833-002	MH645133
19	* Hippocampus severnsi *	Indonesia	AM I.47834-006	MH645134
20	* Hippocampus severnsi *	Indonesia	AM I.47833-003	MH645135
21	* Hippocampus severnsi *	Indonesia	AM I.47834-005	MH645136

#### Diagnosis.

*Hippocampuspontohi* differs from its congeners by the following combination of characters: subdorsal rings 4; two pairs of bilaterally wing-like protrusions formed by a pair of large truncate spines projecting laterad on both first and second superior trunk ridges; laterodorsal surface flat on the third and fourth trunk rings; tail rings 28; dorsal fin rays 12; pectoral fin rays ten.

#### Description.

General body shape as in Figure 6. Morphometric characters listed in Table 1. Head length 21.6% in SL, head depth 64.4% in HL; snout length 24.1% in HL, bulbous tip absent, snout depth 89.0% in SnL; post-orbital 50.9% in HL; distinct, angular coronet, coronet height 46.2% in HL, unbranched dermal appendage attached to anterior part of coronet; single gill-opening on midline behind coronet supported by elevated cleithral ring; dorsal fin 14 rays; pectoral-fin rays nine; anal fin rays four; trunk rings 12, trunk length 33.0% in SL, trunk depth just anterior to dorsal fin base 14.4% in SL; dorsal fin base strongly raised posterodorsad; subdorsal rings four, dorsal fin base starting immediately posterior to ninth trunk ring and ending immediately posterior to first tail ring; no external pouch visible; tail rings 28, tail length 45.3% in SL. Body ornamentation: prominent spine dorsal of eye, small spine ventroposterior to eye; lateral head spine ventral of coronet; two moderately large spines on cleithral ring, upper spine at level of last pectoral fin ray, lower spine at ventral extent of ring; snout spine on midline between eyes; nape spine absent; subdorsal spines four, superior trunk ridge ending with three rounded spines protruding laterad, the posterior spine greatly enlarged on 12^th^ trunk ring; superior trunk ridge with large bilaterally paired truncate spines projecting laterad on first and second trunk rings, laterodorsal surface flat on second, third, and fourth trunk rings, large bilaterally paired spines on fifth trunk ring, and small pair of spines dorsally on sixth trunk ridge; lateral trunk ridge with small spine on fifth trunk ring and large spine on eighth trunk ring; inferior trunk ridge with spines of moderate size beginning on fifth trunk ring and and ending on 11^th^ trunk ring; superior tail ridge spines well developed anteriorly, becoming smaller posteriorly, with enlarged spines on fifth and ninth tail rings; inferior tail ridge spines absent; caudal fin absent.

#### Remarks.

Although *Hippocampuspontohi* was distinguished from *H.severnsi* primarily by features of coloration (Lourie and Kuiter 2008), meristic, morphometric, and diagnostic morphological characters in the original description did not support the separation of these seahorses into two species. The invalidity of *H.severnsi* was subsequently recognised due to the unreliability of employing coloration as a useful diagnostic character in order to distinguish between species of seahorses (Lourie et al. 2016). Here we further support the synonymization of *H.severnsi* under *H.pontohi* based on partial mitochondrial COI genetic data collected from additional 21 vouchered specimens of *H.pontohi* and those referred to as *H.severnsi* (Table 2). Genetic distance analysis (uncorrected p distances) failed to discriminate *H.severnsi* from *H.pontohi* (Suppl. material 1), which revealed an average intraspecific divergence of 0.2%. A neighbour joining tree of the COI sequence data, including COI data from *H.bargibanti*, *H.denise*, and *H.japapigu*, is supplied here as Suppl. material 2.

#### Comparative remarks.

The combination of characters provided in the diagnosis that distinguishes *H.japapigu* from all congeners are presented in Table 3 and summarized below. The new species is unique in *Hippocampus* in possessing a distinct elevated dorsal ridge internally formed by triangular bony mounds in the anterodorsal area of the trunk directly posterior to the head, which we propose as an apomorphy for this species. All currently recognized seahorse species, including the pygmy seahorse congeners, differ in the absence of triangular bony mounds and the presence of typical flat surfaces dorsally on the second to fourth superior trunk rings. *Hippocampusjapapigu* is most similar to *H.pontohi* (Fig. 8, Table 3) in meristics, overall body ornamentation, and the presence of a distinct coronet. They differ primarily on the basis of bilaterally paired wing-like protrusions directly posterior to the head, which are internally formed by a single connected pair of large, truncate spines projecting dorsolaterad on the first superior trunk ridge in *H.japapigu*, as opposed to a double pair of large truncate spines projecting strongly laterad on both the first and second superior trunk ridges in *H.pontohi*. Additional distinctions include patterns of the anterodorsal trunk rings (elevated dorsal ridge formed by triangular bony mounds dorsad on the second to fourth trunk rings in *H.japapigu*, laterodorsal surface flat on the third and fourth trunk rings in *H.pontohi*); eighth lateral trunk ridge spine (very large and prominent spine projecting laterad in *H.japapigu*, small in *H.pontohi*); color pattern (brown with white reticulation, thin red line tracing the superior trunk ridge anterior to dorsal fin base in *H.japapigu*, white, brown, or black color with elliptical markings, each outlined with thin red lines, tracing the entire superior trunk ridge and extending ventrally around the fifth superior and eighth lateral trunk ridge spines in *H.pontohi*). *Hippocampusjapapigu* and *H.pontohi* can be further be distinguished by the number of tail rings (28 vs. 28–30), dorsal fin rays (14 vs. 12), and pectoral fin rays (9 vs. 10).

**Table 3.** Comparison of morphological characters in *Hippocampusjapapigu*, *H.pontohi*, *H.colemani*, *H.satomiae*, and *H.waleananus*.

	* H. japapigu *	* H. pontohi *	* H. colemani *	* H. satomiae *	* H. waleananus *
Voucher number/data source	WA 41200	Lourie and Kuiter 2008	Kuiter 2003	Lourie and Kuiter 2008	Gomon and Kuiter 2009
Single gill opening	present	present	present	present	present
Strongly raised cleithral girdle	present	present	present	present	present
Coronet	distinct and angular	distinct and angular	low and rounded	distinct and angular	low double mound
Cleithral spines	pectoral fin base, ventral	pectoral fin base, ventral	pectoral fin base, ventral	pectoral fin base, ventral	pectoral fin base
Subdorsal rings (3+1)	present	present	present	present	present
Lateral head spine	present	present	present	present	present
Snout spine	present	present	present	present	present
Eye spine dorsal	present	present	present	present(double)	present
Eye spine ventral	present	present	present	absent	present
first superior trunk ridge spines	present	present	present	present	present
second superior trunk ridge spines	absent	present	present	present	†present
Elevated ridge dorsal of trunk	present	absent	absent	absent	absent
fifth superior trunk ridge spines	present	present	present	present	present
fifth lateral trunk ridge spines	present	present	present	present	present
eighth lateral trunk ridge spines (large)	present	present	present	present	present
eighth inferior trunk ridge spines	present	present	present	present	present
12^th^ superior trunk ridge subdorsal spine (large)	present	present	present	present	present
Superior tail ridge spines	5,9,12,16	5,9,12	absent	5,9,12	4,8,12
Inferior tail ridge spines	absent	absent	absent	absent	posterior 28 rings

Several other pygmy seahorse species are morphologically similar to *Hippocampusjapapigu*, including *H.colemani*, *H.satomiae*, and *H.waleananus*. The following characters support the distinctions among these species: number of tail rings (28 in *H.japapigu* vs. 26 in *H.colemani*, 27–28 in *H.satomiae*, 32 in *H.waleananus*); dorsal fin rays (14 in *H.japapigu* vs. 13 in *H.satomiae*, 12 in *H.waleananus*); coronet (distinct in *H.japapigu* vs. low and rounded in *H.colemani*, low double mound in *H.waleananus)*; cleithral ring spines (at pectoral fin base and ventral of head in *H.japapigu* vs. pectoral fin base in *H.waleananus)*; eye spine dorsally (double spine in *H.satomiae*), eye spine ventrally (absent in *H.satomiae*); superior tail ridge spines (fifth, ninth, 12^th^ vs. absent in *H.colemani*, fourth, eighth, 12^th^ in *H.waleananus*); inferior tail ridge spines (absent vs. present on last 28 tail rings in *H.waleananus*).

**Figure 9.**
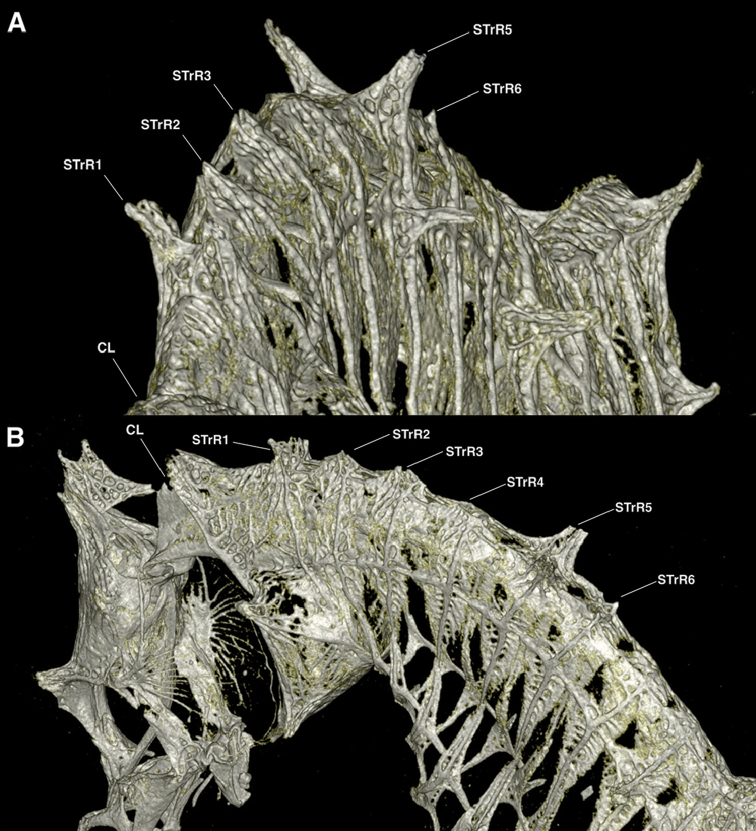
Computed tomography scanned anterior trunk area of *Hippocampusjapapigu*, UW 157506, male holotype, 16.33 mm SL, Hachijo-jima Island, Japan (photograph Graham Short). **A** Anterolateral view **B** Lateral view. Note the pair of spines projecting dorsolaterad on STrR1 and triangular bony mounds arched dorsad on STrR2, STrR3, and STrR4. Abbreviations: CL, cliethral ring; STrR1, first superior trunk ridge; STrR2, second superior trunk ridge; STrR3, third superior trunk ridge; STrR4, fourth superior trunk ridge; STrR5, fifth superior trunk ridge; STrR6, sixth superior trunk ridge.

**Figure 10.**
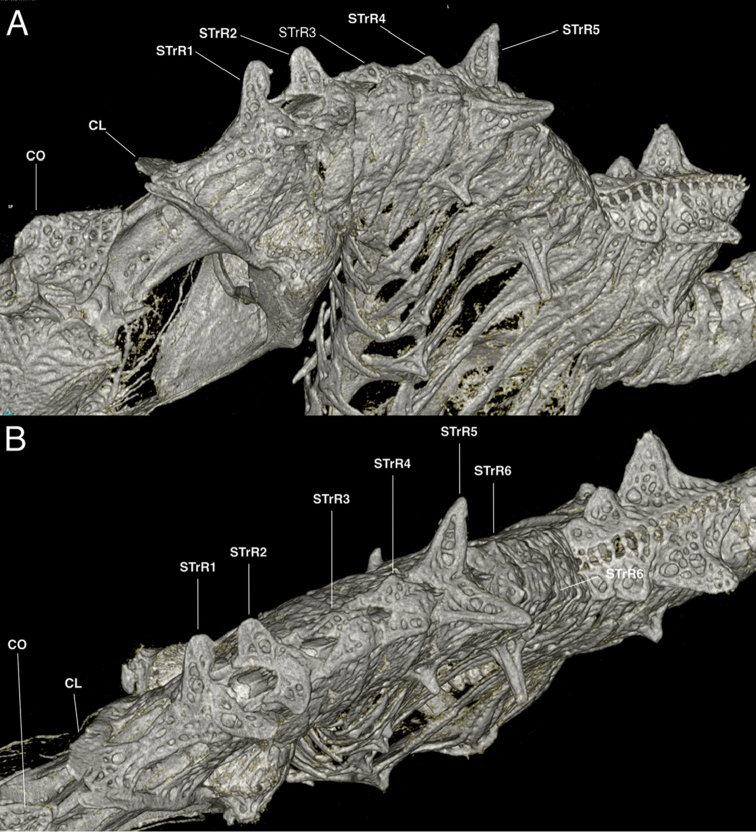
Computed tomography scanned anterior trunk area of *Hippocampuspontohi*, AMS I.47833-001, preserved male non-type, 16.33 mm SL, Cape Kri, Raja Ampat, Indonesia (photograph Graham Short). **A** Anterolateral view **B** Dorsal view. Note the double pair of spines projecting dorsolaterad on STrR1 and STrR1, respectively, and laterodorsal surface flat on STrR3, and STrR4. Abbreviations: CO, coronet; CL, cliethral ring; STrR1, first superior trunk ridge; STrR2, second superior trunk ridge; STrR3, third superior trunk ridge; STrR4, fourth superior trunk ridge; STrR5, fifth superior trunk ridge; STrR6, sixth superior trunk ridge.

*Hippocampusjapapigu* shares with *H.pontohi*, *H.colemani, H.satomiae*, and *H.waleananus* 12 trunk rings, strongly raised continuous cleithral ring, and presence of diagnostic body ornamentation (snout spine, eye spines, two cliethral spines, lateral head spine, large spine on fifth superior trunk ridge, large spine on eighth lateral trunk ridge, small spine on fifth lateral trunk ridge, Table 2), including wing-like-protrusions immediately posterior to the head. Based on careful visual examinations of *in situ* underwater photographs, x-rays, and type material (Kuiter 2003; Lourie and Kuiter 2008; Gomon and Kuiter 2009; Smith 2017), it appears *H.japapigu* shares with *H.waleananus* a single pair of bilaterally paired wing-like protrusions (vs. double pair in *H.colemani* and *H.satomiae)*. In the original description of *H.colemani* (Kuiter, 2003), the number of trunk rings was diagnosed as 11, however in our comparative analysis of trunk ring counts, we detected the presence of 12 trunk rings in the x-ray of the holotype of *H.colemani* (Gomon & Kuiter, 2009, AMS I.41181-001, fig. 3B). Furthermore, we noted 4 subdorsal rings (three trunk and one tail rings) via μCT in *H.japapigu* and the non-type *H.pontohi*, and similarly four subdorsal rings in our examination of the radiographs of *H.colemani*, *H.pontohi*, and *H.satomiae* (Kuiter 2003, fig. 3B; Lourie and Kuiter 2008, figs 2A,C; Gomon and Kuiter 2009, fig. 3B). In contrast, three subdorsal trunk rings were noted in the original diagnoses of these pygmy seahorses. *Hippocampusjapapigu* and *H.pontohi* retain the ring and ridge structure of larger seahorses, and with μCT scans, we can detect well-developed ossification of the skeleton, including the strong ossification of the inferior and ventral trunk area (Figs 8–10). Similarly, Gomon & Kuiter (2009, fig. 3B) detected a well-formed skeleton in *H.colemani* via x-ray. In contrast, *H.bargibanti* and *H.denise* reveal incomplete ossification of the inferior and ventral trunk ridges anteriorly, the ridges reduced to star-shaped dermal ossifications (Gomon 1997; Lourie and Randall 2003; Gomon and Kuiter 2009).

#### Genetic comparisons.

Suppl. material 1 shows genetic distance analysis at the COI gene (uncorrected p distances) between *H.japapigu* and previously sequenced non-type specimens of the pygmy seahorses *H.pontohi*, *H.bargibanti*, and *H.denise* (Hamilton et al. 2017), and additional vouchered specimens of *H.pontohi*. *Hippocampusjapapigu* differs from *H.pontohi* by 10.1%, from *H.bargibanti* by 13.0%, and *H.denise* by 10.1%. Reported mtDNA clock rates of approximately 1.2% per million years in marine teleosts (Reece et al. 2010) indicate divergence between *H.japapigu* and *H.pontohi* approximately 8.4 million years ago.

#### Distribution and habitat.

*Hippocampusjapapigu* sp. n. is only known to occur in Japan, from scattered localities including Kashiwa-jima Island, Sukumo Bay; Kushimoto, Kii Peninsula; Osezaki, Izu Peninsula; the Izu Islands of Miyake and Hachijo; Sagami Bay; and Chichi-jima, Ogasawara Islands. The specimens described herein were found off the northwest coast of Hachijo-jima Island at a depth of 10–13 m, and have been anecdotally reported elsewhere at 5–22 m by local divers. Owing to its diminutive size and extraordinary crypsis, this species may have a wider distribution within Japan. The new taxon is not associated with a particular host, and has been observed in association with mixed soft coral, the coralline algae *Halimeda* sp., and hydroids on rocky reef walls and large boulders in both exposed and semi-sheltered locations. During 15 dives initially spent searching ad hoc for this species by the second author in July 2013, 13 individuals were observed in an approximately 100 m stretch of rocky reef. These ranged in depth from 10 to 20 m and water temperature fluctuated between 19–24°C over 6 days. When one individual was discovered, another was often found in close proximity and appeared to represent male-female pairs. Returning in June 2015 with a larger group of experienced dive guides, with 10 dives searching for the species, only a single individual was found, possibly suggesting fluctuations in the abundance of the species. Several pregnant males were observed in July 2013, but it is unknown whether reproduction occurs seasonally or year-round.

#### Etymology.

The specific epithet is from the colloquial Japanese name of the new species, Japan Pig, Japapigu, or 日本のピグミータツノオトシゴ.

#### Common name.

New common English and Japanese names, Japanese Pygmy Seahorse and Hachijo-tatsu, respectively, are proposed here for *Hippocampusjapapigu*.

## Discussion

Here we consider *Hippocampusjapapigu* as a valid species due to its morphological uniqueness; however, a more detailed phylogenetic and systematic study is necessary to understand its evolutionary relationship to other pygmy seahorses. Using μCT, we have identified key diagnostic characters in the anterodorsal area of the trunk that differentiate *H.japapigu* from the morphologically similar *H.pontohi*. Unequivocally, the most noticeable skeletal characters of *H.japapigu* are the unusual triangular bony mounds that serve as a structural basis for the elevated dorsal ridge along the trunk, and the presence of a single pair of large truncate and connected spines projecting dorsolaterally of the trunk that form the bilateral wing-like protrusions behind the head. In the previous diagnoses of *H.colemani*, *H.pontohi*, *H.satomiae* and *H.waleananus*, these dorsolateral truncate spines were difficult to discern via traditional photography and radiographs (Kuiter 2003; Lourie and Kuiter 2008; Gomon and Kuiter 2009). In *H.colemani* (Kuiter 2003) these spines were not noted; in *H.pontohi* (Lourie and Kuiter 2008, fig. 2A) these spines were described as dorsolateral expansions of the first and second superior trunk rings with no mention of the presence of spines; in *H.satomiae* (Lourie and Kuiter 2008, fig. 2C) these spines were diagnosed as fused spines on the first and second superior trunk ridges, however, in the radiograph provided in the description they appear to be two pairs of spines, and separated; in *H.waleananus* (Gomon and Kuiter 2009, fig. 3A) these spines were described as wing-like protuberances on the second superior trunk ridge with no diagnosis of spines. In the latter, we detect a pair of large spines on the first superior trunk ridge (Gomon and Kuiter 2009, fig. 3A). Additional characters that were not detected via microscopy or high-resolution photography include a small pair of spines dorsally on the sixth superior trunk ridge in both *H.japapigu* and *H.pontohi* (Figs 8–10). Therefore, μCT offers new avenues for enhancing taxonomic descriptions by documenting otherwise difficult or indiscernible diagnostic skeletal features in small specimens.

*Hippocampusjapapigu* is known to occur throughout subtropical southeast Japan where investigations of inshore marine ichthyofauna (Senou et al. 2006) have recorded similar species compositions between southern Honshu and the Izu and Ogasawara Islands, suggesting a passive and long distance recruitment and dispersal due to the influence of the Kuroshio Current (Kuriiwa et al. 2014). This north-flowing ocean current, which originates east of the Philippine coast, flows alongside Taiwan to the south coast of the major islands of Japan, including Honshu, and extends northward and southward between the Izu and Ogasawara Islands, is likely to act as an important conduit to transport tropical fishes from the south to these islands. *Hippocampusjapapigu* has not been observed in the Ryukyu Islands to date despite these islands being located in the Kuroshio Current. Given time, scuba divers may observe the new species in the Ryukyu Islands, and even as far south as Taiwan where *H.bargibanti* (Kwang-Tsao et al. 2008), *H.colemani*, and a species appearing to be *H.pontohi* have been observed by local scuba divers.

## Supplementary Material

XML Treatment for Hippocampus japapigu

XML Treatment for Hippocampus pontohi

## Supplementary materials

Supplementary material 1 Genetic distance analysis (uncorrected p distances) of COI sequence data from 21 specimens of *H.pontohi* and those referred to *H.severnsi* Data type: molecular data This is an open access article distributed under the terms of the Creative Commons Attribution License (CC BY 4.0), which permits unrestricted use, distribution, and reproduction in any medium, provided the original author and source are credited.Graham Short, Richard Smith, Hiroyuki Motomura, David Harasti, Healy Hamilton

Supplementary material 2 NJ tree of COI sequences from 21 specimens of *H.pontohi* and those referred to *H.severnsi* Data type: phylogenetic tree This is an open access article distributed under the terms of the Creative Commons Attribution License (CC BY 4.0), which permits unrestricted use, distribution, and reproduction in any medium, provided the original author and source are credited.Graham Short, Richard Smith, Hiroyuki Motomura, David Harasti, Healy Hamilton
