# Supplementary figures and images for: Hippocampusjapapigu, a new species of pygmy seahorse from Japan, with a redescription of H.pontohi (Teleostei, Syngnathidae)
Source: Zookeys. 2018 Aug 2;(779):27–49. doi: 10.3897/zookeys.779.24799 (PMC6110155; doi:10.3897/zookeys.779.24799)

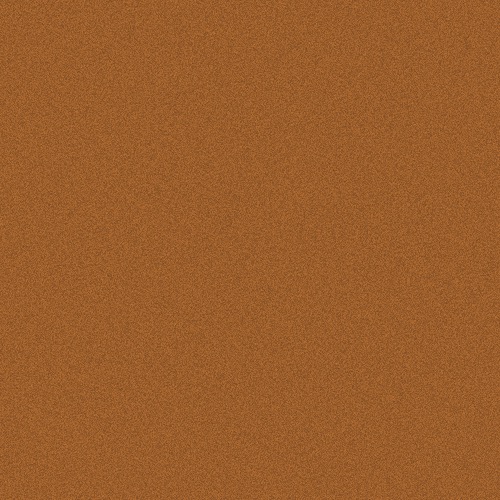

Supplement: Supplementary material 1 — Genetic distance analysis (uncorrected p distances) of COI sequence data from 21 specimens of H.pontohi and those referred to H.severnsi [file zookeys-779-027-s001.xls › Data/PresetImageFill5-15.jpg]

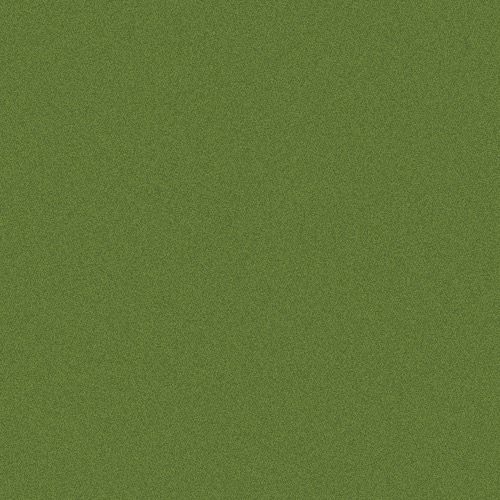

Supplement: Supplementary material 1 — Genetic distance analysis (uncorrected p distances) of COI sequence data from 21 specimens of H.pontohi and those referred to H.severnsi [file zookeys-779-027-s001.xls › Data/PresetImageFill2-12.jpg]

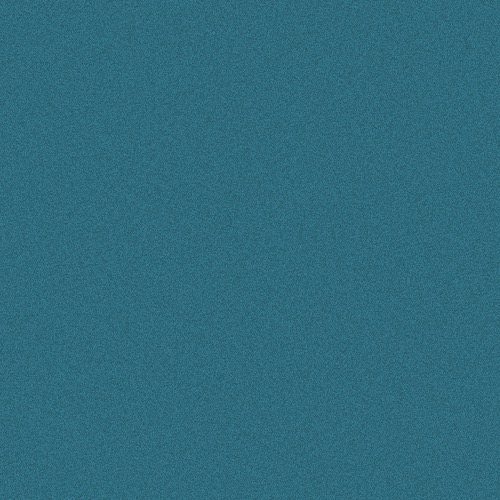

Supplement: Supplementary material 1 — Genetic distance analysis (uncorrected p distances) of COI sequence data from 21 specimens of H.pontohi and those referred to H.severnsi [file zookeys-779-027-s001.xls › Data/PresetImageFill4-14.jpg]

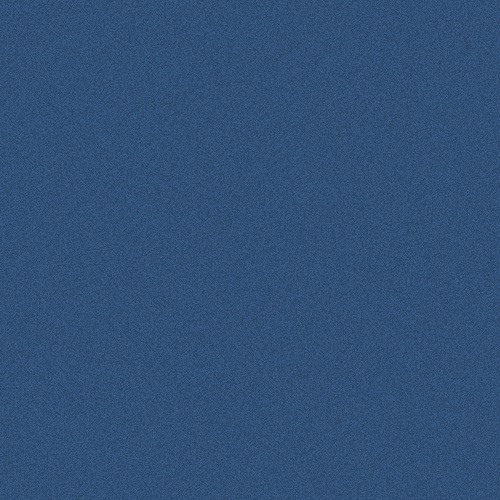

Supplement: Supplementary material 1 — Genetic distance analysis (uncorrected p distances) of COI sequence data from 21 specimens of H.pontohi and those referred to H.severnsi [file zookeys-779-027-s001.xls › Data/PresetImageFill0-10.jpg]

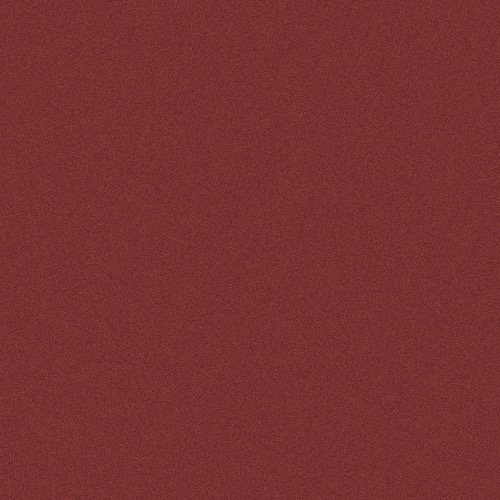

Supplement: Supplementary material 1 — Genetic distance analysis (uncorrected p distances) of COI sequence data from 21 specimens of H.pontohi and those referred to H.severnsi [file zookeys-779-027-s001.xls › Data/PresetImageFill1-11.jpg]

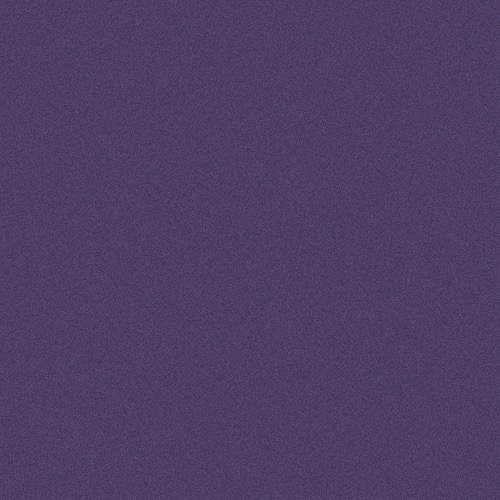

Supplement: Supplementary material 1 — Genetic distance analysis (uncorrected p distances) of COI sequence data from 21 specimens of H.pontohi and those referred to H.severnsi [file zookeys-779-027-s001.xls › Data/PresetImageFill3-13.jpg]

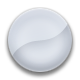

Supplement: Supplementary material 1 — Genetic distance analysis (uncorrected p distances) of COI sequence data from 21 specimens of H.pontohi and those referred to H.severnsi [file zookeys-779-027-s001.xls › Data/bullet_gbutton_gray-16.png]

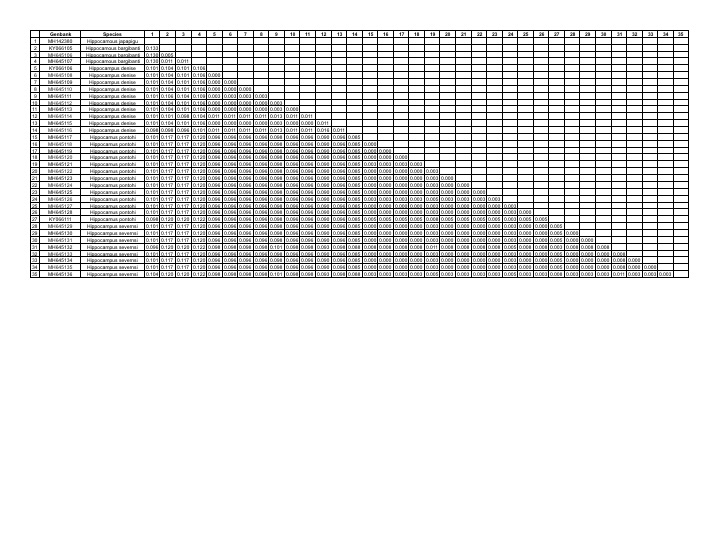

Supplement: Supplementary material 1 — Genetic distance analysis (uncorrected p distances) of COI sequence data from 21 specimens of H.pontohi and those referred to H.severnsi [file zookeys-779-027-s001.xls › preview.jpg]

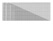

Supplement: Supplementary material 1 — Genetic distance analysis (uncorrected p distances) of COI sequence data from 21 specimens of H.pontohi and those referred to H.severnsi [file zookeys-779-027-s001.xls › preview-micro.jpg]

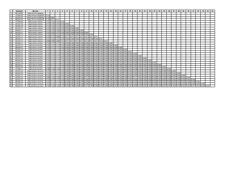

Supplement: Supplementary material 1 — Genetic distance analysis (uncorrected p distances) of COI sequence data from 21 specimens of H.pontohi and those referred to H.severnsi [file zookeys-779-027-s001.xls › preview-web.jpg]

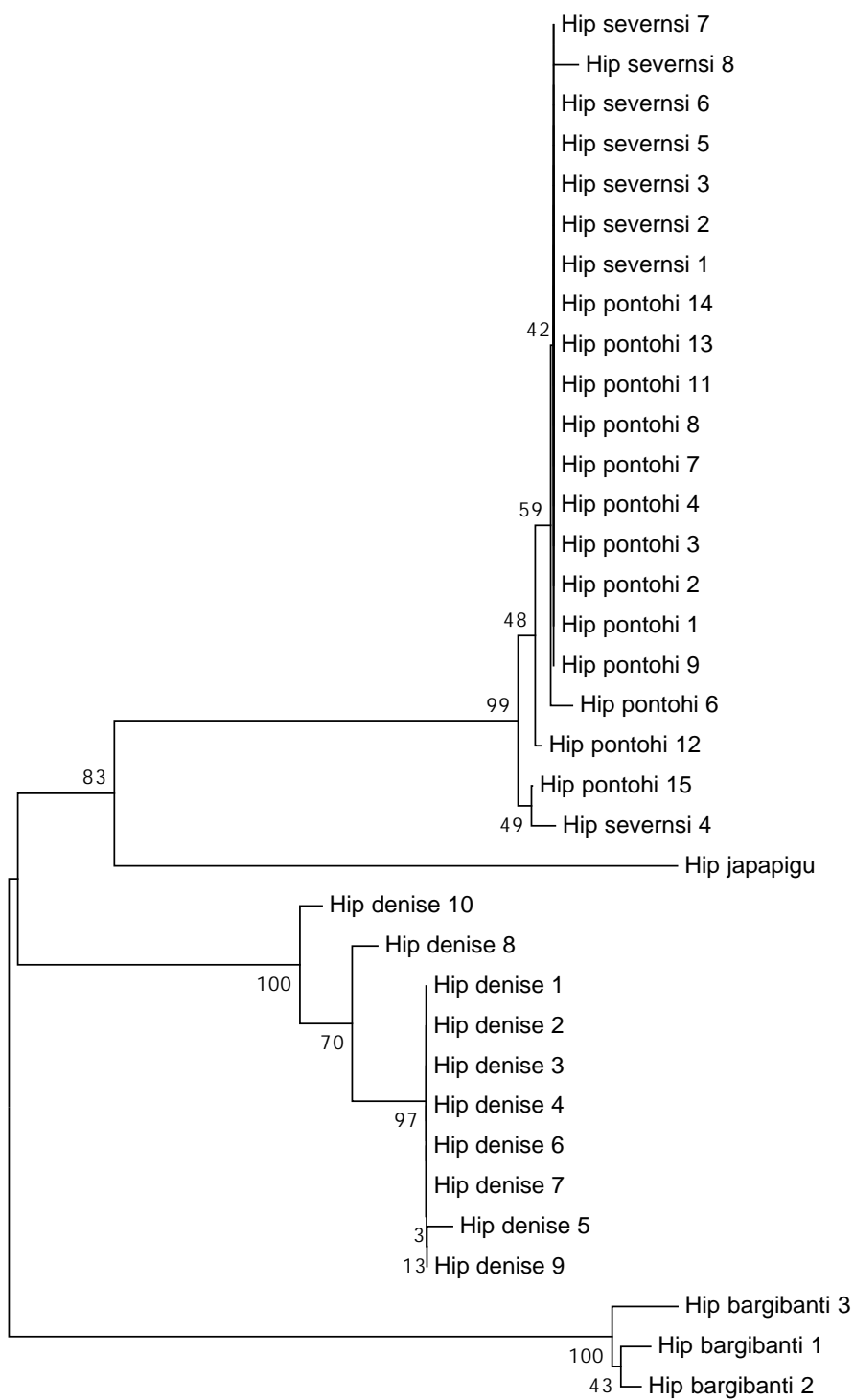

0.010

Supplement: Supplementary material 2 — NJ tree of COI sequences from 21 specimens of H.pontohi and those referred to H.severnsi [file zookeys-779-027-s002.pdf]
